# Melittin—A Natural Peptide from Bee Venom Which Induces Apoptosis in Human Leukaemia Cells

**DOI:** 10.3390/biom10020247

**Published:** 2020-02-06

**Authors:** Michal Ceremuga, Maksymilian Stela, Edyta Janik, Leslaw Gorniak, Ewelina Synowiec, Tomasz Sliwinski, Przemyslaw Sitarek, Joanna Saluk-Bijak, Michal Bijak

**Affiliations:** 1 Military Institute of Armament Technology, Prymasa Stefana Wyszyńskiego 7, 05-220 Zielonka, Poland; ceremugam@witu.mil.pl; 2 CBRN Reconnaissance and Decontamination Department, Military Institute of Chemistry and Radiometry, Antoniego Chrusciela “Montera” 105, 00-910 Warsaw, Poland; m.stela@wichir.waw.pl; 3 Biohazard Prevention Centre, Faculty of Biology and Environmental Protection, University of Lodz, Pomorska 141/143, 90-236 Lodz, Poland; edyta.janik@unilodz.eu (E.J.); lmgorniak@gmail.com (L.G.); 4 Laboratory of Medical Genetics, Faculty of Biology and Environmental Protection, University of Lodz, Pomorska 141/143, 90-236 Lodz, Poland; ewelina.synowiec@biol.uni.lodz.pl (E.S.); tomasz.sliwinski@biol.uni.lodz.pl (T.S.); 5 Department of Biology and Pharmaceutical Botany, Medical University of Lodz, 90-151 Lodz, Poland; przemyslaw.sitarek@umed.lodz.pl; 6 Department of General Biochemistry, Faculty of Biology and Environmental Protection, University of Lodz, Pomorska 141/143, 90-236 Lodz, Poland; joanna.saluk@biol.uni.lodz.pl

**Keywords:** melittin, bee venom, apoptosis, leukaemia

## Abstract

Bee venom is a very complex mixture produced and secreted by the honeybee (*Apis mellifera*). Melittin is a major component of bee venom that accounts for about 52% of its dry mass. A vast number of studies have been dedicated to the effects of melittin’s regulation of apoptosis and to the factors that induce apoptosis in various types of cancer such as breast, ovarian, prostate, lung. The latest evidence indicates its potential as a therapeutic agent in the treatment of leukaemia. The aim of our present study is to evaluate melittin’s ability to induce apoptosis in leukaemia cell lines of different origin acute lymphoblastic leukaemia (CCRF-CEM) and chronic myelogenous leukaemia (K-562). We demonstrated that melittin strongly reduced cell viability in both leukaemia cell lines but not in physiological peripheral blood mononuclear cells (PMBCs). Subsequent estimated parameters (mitochondrial membrane potential, Annexin V binding and Caspases 3/7 activity) clearly demonstrated that melittin induced apoptosis in leukaemia cells. This is a very important step for research into the development of new potential anti-leukaemia as well as anticancer therapies. Further analyses on the molecular level have been also planned (analysis of proapoptotic genes expression and DNA damages) for our next research project, which will also focus on melittin.

## 1. Introduction

Honeybees (*Apis mellifera*) produce and secrete a venom that has complex structure. The injection of bee venom into specific points on a body, a method which has been widely used as a complementary and alternative therapy for 3000 years, is called a bee venom therapy [1]. The composition of bee venom is very diverse. The vast majority are peptides: melittin, apamin, MCDP peptide, tertiapine, secapoin, adolapine, prokamine and proteins with enzymatic properties: phospholipase A2, hyaluronidase, phosphomonoesterase, lysophospholipase, α-d-glucosidase. In addition, honeybee venom contains many low molecular mass compounds such as sugars, amino acids, phospholipids, pheromones and biogenic amines [2]. Extensive research conducted in recent years has shown that satisfactory results may be achieved when using bee venom for various types of diseases such as arthritis [3], chronic pain [4], cancer [5] and atopic dermatitis [6].

**Figure 1:**
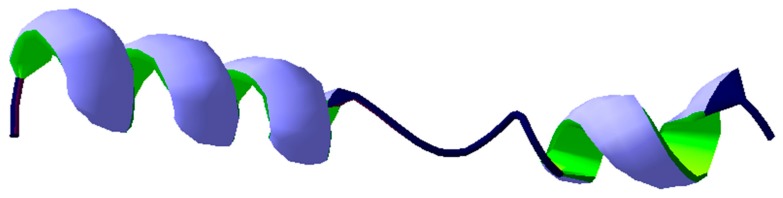
The 3D structure of melittin (PDB: 2MW6 was downloaded from the RCSB PDB databank (http://www.rcsb.org/) [13]). The visualisation was rendered using the Swiss-PdbViewer [14].

The major component of bee venom is called melittin. This accounts for about 52% of dry mass. It contains 26 amino polypeptides and has a molecular mass of 2840 Da. This peptide is synthesised in a bee’s venom gland in the form of an inactive precursor known as ’prepromelittine’, which consists of 70 amino acids. The activation of prepromelittin is a multistep process. First, a predominantly hydrophobic 21 amino acid signal peptide is cleaved, second, a carboxy-terminal amide is formed with an associated loss of the terminal glycine followed by the removal of a 22 amino acid fragment with proline or alanine at even-numbered positions, by an exoprotease [7]. The active form of melittin possesses polar properties where the first 20 amino acids (from N-terminus) are hydrophobic while the next 6 one are hydrophilic. The following is the entire sequence: NH_2_-Gly-Ile-Gly-Ala-Val-Leu-Lys-Val-Leu-Thr-Thr-Gly-Leu-Pro-Ala-Leu-Ile-Ser-Trp-Ile-Lys-Arg-Lys-Arg-Gln-Gln-CONH_2_ [8]. This specific sequence occurs because melittin has a very unique 3-dimensional structure [9,10]. The N-terminal region has +4 charges, +2 charges are at C terminal which makes a total of +6 charges at physiological pH [11]. Under normal physiological conditions, melittin forms a random coil, which when bound to the lipid bilayer of the cell membrane, spontaneously forms a monomeric alpha-helix. The melittin monomer consists of 2 helices joined by a coiled region containing a proline residue. As a result, this peptide is able to penetrate the cell membrane and molecularly act on cellular sub-structures [12]. The 3D structure of melittin is shown below in Figure 1.

In addition to being the major compound present in bee venom, melittin is also the most bioactive component of this mixture. Pure melittin comprises a broad spectrum of biological activities that can be utilised in modern medical therapies. In 1985, Hait et al. demonstrated that melittin is able to inhibit clonogenicity and the growth of leukemic cells in humans and mice and its potency derives from the action of the ubiquitous calcium-binding protein–calmodulin [15]. Since then, the mechanism and functioning of melittin-induced apoptosis have been extensively studied in numerous cancer cell lines [5]. It has also been observed that melittin, in a synergistic manner, increases the toxicity of bleomycin toward human granulocytes and macrophages as well as red blood stem cells [16]. The examples of various biological effects of melittin are shown in Table 1.

The aim of our study is the evaluation of the ability of melittin to induce apoptosis in leukaemia cell lines of two different origins, acute lymphoblastic leukaemia and acute promyelocytic leukaemia. The main studies were focused on establishing LC_50_ parameters, confirming the apoptosis process in leukaemia cells as well as on the verification of the effect of melittin on healthy peripheral blood mononuclear cells (PBMC).

## 2. Materials and Methods

### 2.1. Reagents

Dimethyl sulfoxide (DMSO), Tris, Histopaque^®^-1077, RPMI 1640, (S)-(+)-camptothecin (C9911) and melittin from honeybee venom (cat. No. M2272) were from the Sigma-Aldrich Chemical Co. (St. Louis, MO, USA). Cell viability kit including cell counter slides and trypan blue dye was obtained from BIO-RAD (Hercules, CA, USA). Penicillin–streptomycin mixture, heat-inactivated fetal bovine serum (FBS), phosphate-buffered saline (PBS) without calcium and magnesium were obtained in Lonza (Basel, Switzerland). The JC-1 Dye, 3-(4,5-dimethylthiazol-2-yl)-2,5-diphenyltetrazolium bromide—MTT, Hank’s Balanced Salt Solution (HBSS) and CellEvent™ Caspase-3/7 Green Flow Cytometry Assay Kit were from Thermo Fisher Scientific (Waltham, MA, USA). FITC stained Annexin V Apoptosis Detection Kit I was obtained from Becton Dickinson (Franklin Lakes, NJ, USA). All other chemicals were of molecular grade.

### 2.2. Cellular Material

In this study, the following two human leukaemia cell lines were used: acute lymphoblastic leukaemia CCRF-CEM (CCL-119, ATCC™) and chronic myelogenous leukaemia K-562 (CCL-243, ATCC™). Both cell lines were obtained from the American Type Culture Collection (ATCC™, Manassas, VA, USA).

Additional lines were health peripheral blood mononuclear cells obtained from whole blood samples which were collected from healthy donors in the Regional Centre for Transfusion Medicine in Lodz (Poland). The blood samples collection procedure in the morning (between 8 to 10 am) from fasting donors was performed after a medical examination to ensure that there were no cardiovascular disorders, allergies, lipid or carbohydrate metabolism disorders as well as allergy [33]. A PBMC fraction was isolated using density-gradient centrifugation in a Histopaque^®^-1077 (a sterile solution of polysucrose, 57 g/L, and sodium diatrizoate, 90 g/L, with a density of 1.077 g/mL) solution. After a maximum of two hours from the time of collection, blood was carefully layered (in a volume ratio of 1:1) onto the Histopaque^®^-1077, and centrifuged for 30 min (400× *g*, RT). Afterwards, the collected pellet was washed twice with RPMI 1640 (400× *g*, 10 min, RT).

### 2.3. Cell Cultures

All cell lines were cultured with the same protocol in RMPI 1640 medium with 100 units of penicillin and 100 μg of streptomycin sulphate per 1 mL of culture media supplemented with 10% (*v*/*v*) FBS. The cell growing process was performed in a humidified incubator at 37 °C and 5% CO_2_. To perform the analysis cells were seeded at 3 × 10^6^ cells per well and were left in the incubator for 12 h before treatment procedures [34]. Next, the cell samples were incubated with melittin in a concentration range of 0.001 to 100 µM for 24 h and 48 h. As a positive control (S)-(+)-camptothecin was used in three different concentration—10, 50 and 100 µM.

### 2.4. Cell Viability Determination

The viability of cells treated by melittin was evaluated by two different and independent methods.

The first was based on a trypan blue dye method using BIO-RAD TC20 automated cell counter (Hercules, CA, USA) according to the manufacturer’s protocol. The cell viability was expressed as a percentage relative to the untreated (control) cells, defined as 100%.

The second method was based on MTT assay. MTT solution—0.5 mg/mL was added to all samples and incubated at 37 °C. After 4 h, the MTT solution was discarded carefully, and the formed formazan crystals were dissolved in DMSO. The amount of formed formazan crystals was measured colorimetrically at a wavelength of 570 nm with background subtraction at 630 nm on a Microplate Reader—BioTek Synergy HT (BioTek Instruments, Winooski, VT, USA). Cell viability was expressed as a percentage relative to the untreated (control) cells, defined as 100%.

### 2.5. Mitochondrial Membrane Potential (MMP)

The mitochondrial membrane potential (MMP) is one of the most important parameters used for evaluating mitochondrial function—a key indicator of cell health. In this analysis, we used cell membrane-permeable fluorescent dyes—JC-1 (5′,6,6′-tetrachloro-1,1′,3,3′-tetraethylbenzimidazolylcarbocyanine iodide). In the first step, cells were seeded into 96-well black plates with transparent bottoms dedicated to fluorescence measurements (Greiner Bio-One, Kremsmünster, Austria) at a density of 1 × 10^5^ cells/well in 50 μL culture medium and allowed to adhere for 12 h. Next, the cells were incubated with melittin according to the procedure described in the previous point. After treatment, cells were preincubated for 30 min with 5 μM JC-1 dye (prepared in the HBBS) at 37 °C in a CO_2_ (5%) incubator. Finally, cells were centrifuged (300× *g* for 10 min at 22 °C) and washed twice with the HBSS. The fluorescence was measured on a Bio-Tek Synergy HT Microplate Reader (Bio-Tek Instruments, Winooski, VT, USA), with filter pairs of 530 nm/590 nm and 485 nm/538 nm. Results have been presented as a ratio of fluorescence, measured at 530 nm/590 nm to that measured at 485 nm/538 nm (aggregates to monomer fluorescence).

### 2.6. Apoptosis Assay—Anexin V Binding

The presence of apoptotic cells was evaluated based on the exposition of phosphatidylserine residues by the flow cytometry method using FITC Annexin V Apoptosis Detection Kit I. After the culturing procedure, the cells were washed twice in cold PBS and suspended in 100 µL 1× annexin-binding buffer (cells density 1 × 10^5^) in the cytometric tube. Next, 5 µL of FITC stained Annexin V and 5 µL of PI was added and samples were incubated at 37 °C in an atmosphere of 5% CO_2_ for 15 min. After the incubation period, 400 μL of the 1x annexin-binding buffer was added and samples were analysed using the flow cytometer PARTEC CUBE 6 (Görlitz, Germany) at 488-nm excitation wave. Gates for PI (630 nm Longpass filter) and FITC (536/40 nm filter) fluorescence’s were determined according to the fluorescence level of unstained probes. The apoptotic index was calculated as the mean percentage of FITC positive cells in 5 × 10^4^ cells measured in each experiment. Data analysis from experiments was performed in CyFlow version 1.5.1.2. The established gates are presented on Figure S1 in the Supplementary Material.

### 2.7. Determining the Activity of Caspase-3/Caspase-7

To determine the activity of caspase-3/caspase-7, CellEvent™ Caspase-3/7 Green Flow Cytometry Assay Kit was used. After the culturing procedure, the cells were washed twice in cold PBS and suspended (cells density 1 × 10^5^) in 1 mL PBS in a cytometric tube. Next, 1 µL of CellEvent™ Caspase-3/7 Green Detection Reagent was added and mixed gently. After a 30-min incubation period at 37 °C in an atmosphere of 5% CO_2_, 1 µL of the 1 mM SYTOX™ AADvanced™ (prepared in DMSO) was added and left in dark, RT for 5 min. Analysis of the samples without washing or fixing was performed using the Bio-Tek Synergy HT Microplate Reader (Bio-Tek Instruments, Winooski, VT, USA), with filter pairs of 530 nm/590 nm and 485 nm/538 nm.

### 2.8. Data Analysis

All the experimental values obtained were elaborated using Microsoft Excel (Redmond, WA, USA) and expressed as mean ± standard deviation (SD). The statistical analysis was performed using StatsDirect statistical software V. 2.7.2. (Cheshire, UK). In the first step, all results were analysed according to the normality of the distribution by the Shapiro-Wilk test. Next, the results were analysed according to equality of variance via Levene’s test. The significance of the differences between the values was analysed using ANOVA, Tukey’s range test (for data with normal distribution and equality of variance) or the Kruskal–Wallis test; *p* < 0.05 was accepted as statistically significant [35,36,37].

## 3. Results

**Figure 2:**
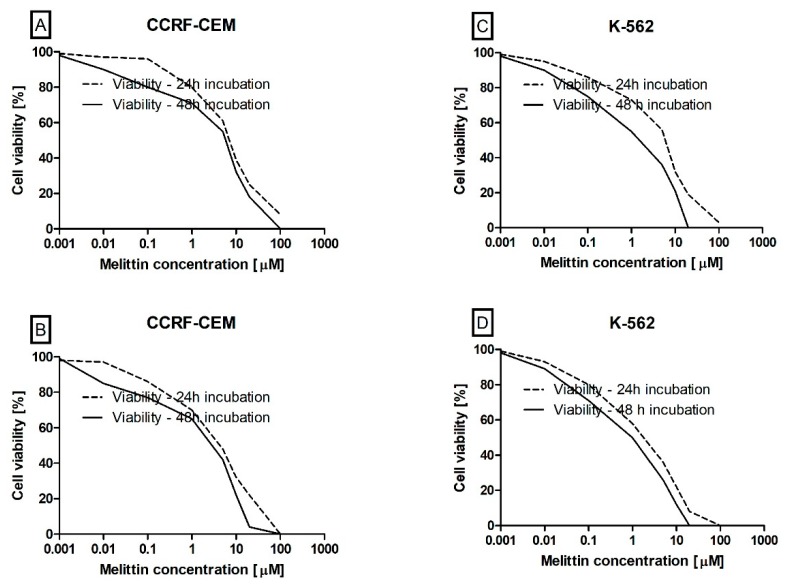
The effect of melittin (in range concentration range 0.001 to 100 µM) on CCRF-CEM (**A**,**B**) and K-562 (**C**,**D**) cells viability. Cell viability was estimated by using trypan blue (**A**,**C**) and MTT (**B**,**D**) methods. The data represents cell viability curves obtained from four measurements (*n* = 4).

The primary aim of our study was to demonstrate the effect of melittin on cell viability of acute lymphoblastic leukaemia (CCRF-CEM) and chronic myelogenous leukaemia (K-562) cell lines. During the cytotoxicity assays in both methods used (trypan blue and MTT), we observed dose dependent cytotoxic effects of melittin on all tested leukaemia cells. This effect has been presented on the cytotoxicity curves (Figure 2). Based on the calculation obtained from these curves, we estimated the lethal concentration 50 (LC_50_) parameters, which for the trypan blue method (Figure 2A,C) were as follows: 7.5 µM—24 h incubation of CCRF-CEM, 6.1 µM—48 h incubation of CCRF-CEM, 5.6 µM—24 h incubation of K-562 and 2.05 µM—48 h incubation of K-562. The same parameter has been also calculated for the second cell viability method—MTT (Figure 2B,D) these were: 4.65 µM—24 h incubation of CCRF-CEM; and 3.6 µM—48 h incubation of CCRF-CEM; 2.45 µM—24 h incubation of K-562; and 1 µM—48 h incubation of K-562.

**Figure 3:**
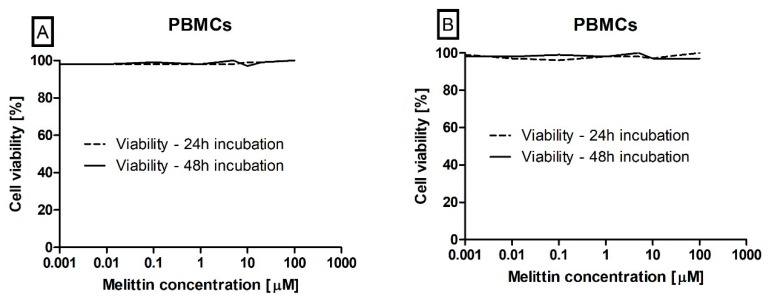
The effect of melittin (in range concentration range 0.001 to 100 µM) on peripheral blood mononuclear cells (PMBCs) viability. Cell viability has been estimated by using both the trypan blue (**A**) and MTT (3-(4,5-dimethylthiazol-2-yl)-2,5-diphenyltetrazolium bromide) (**B**) methods. The data represents cell viability curves obtained by making three measurements (*n* = 3).

Next, we assessed the potential cytotoxicity of melittin using a model of PBMCs that was cultured under the same conditions as the leukaemia cells. Melittin does not cause toxicity in PMBCs as no statistically significant difference was observed in cell viability, as measured by trypan blue and MTT, when incubated with the toxin at 0.001–100 μM (Figure 3A,B).

**Figure 4:**
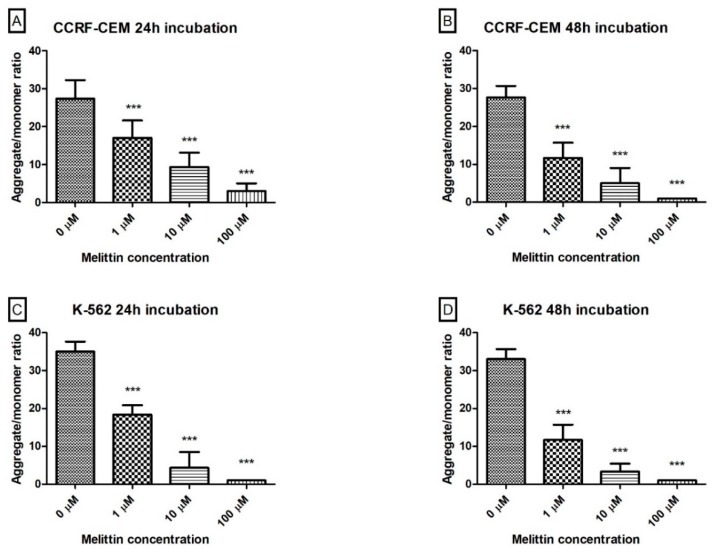
The effect of melittin on mitochondrial membrane potential (MMP) in CCRF-CEM (**A**,**B**) and K-562 (**C**,**D**) cells. Fluorescence was measured after a 24 h (**A**,**C**) and 48 h (**B**,**D**) incubation. Mitochondria in cells are polarized and JC-1 accumulates in mitochondria as an aggregate with red fluorescence, while in apoptotic cells, JC-1 remains in the cytoplasm in a monomeric form emitting green fluorescence. Values are means ± SD (*n* = 4). *** *p* < 0.001.

Mitochondrial dysfunction has been shown to be a contributory factor in the induction of apoptosis and has even been suggested to be central to the apoptotic pathway. Depolarization of the transmembrane potential (Δψm), releasing the apoptogenic factors and loss of oxidative phosphorylation are one of the major observed events in the induction of apoptosis. The ratio of red/green fluorescence (aggregates to monomer fluorescence) of used JC-1 dye is dependent only on membrane potential and is not influenced by other characteristics of the mitochondria such as its shape, size, or density. During our experimental procedure, we observed that melittin induced a potent loss in ΛΨm after both 24-h and 48-h treatments in both tested cell lines (CCRF-CEM and K-562) at all tested concentrations. Compared to controls, the treated cells displayed a statistically significant (*p* < 0.001) decrease in mitochondrial membrane potential as measured by the ratio of JC-1 aggregate/monomer (Figure 4).

**Figure 5:**
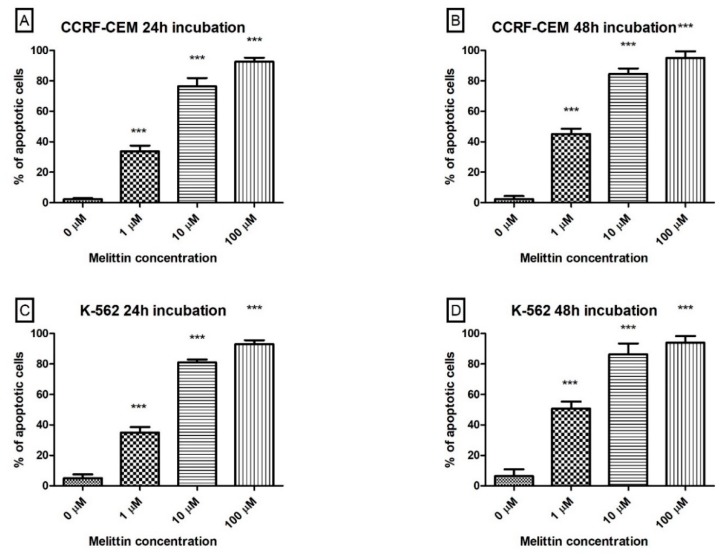
The effect of melittin on apoptosis induction in CCRF-CEM (**A**,**B**) and K-562 (**C**,**D**) cells. Apoptosis was assayed by flow cytometry with Annexin V-propidium iodide staining after 24 h (**A**,**C**) and 48 h (**B**,**D**) incubation with toxin. Values are means ± SD (*n* = 4). *** *p* < 0.001.

The next step focused on confirmation of the apoptotic process in a leukaemia cell after melittin treatment. The effects of melittin on CCRF CEM and K-562 cells apoptosis were investigated by using the flow cytometry method using simultaneous staining with propidium iodide and Annexin V. By using the double-staining flow cytometry method, we observed that incubation of leukaemia cells with melittin results in a dose- and time-dependent increase of Annexin V fluorescence which was related to the apoptosis process of cells. In Figure 5, we presented the results obtained during this analysis. In the highest tested concentration of toxin—100 µM the % of Annexin V stained CCRF CEM cells was 92.5% after 24 h of incubation (Figure 5A) and 95% after 48 h of incubation (Figure 5B). In the case of K-562 the results obtained were similar and were 93% after 24 h of incubation (Figure 5C) and 94% after 48 h of incubation (Figure 5D). In positive control, almost 100% apoptosis was observed in (S)-(+)-camptothecin at the concentration of 50 µM.

**Figure 6:**
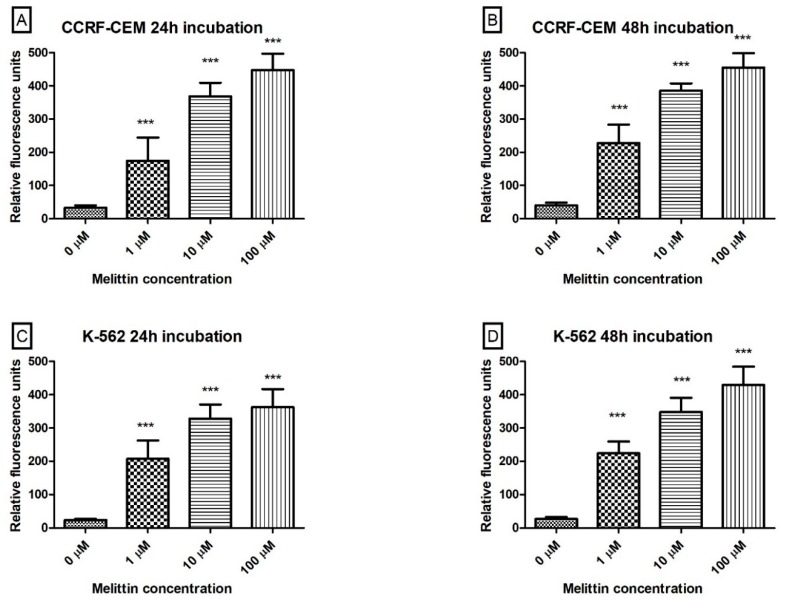
The effect of melittin on caspases 3/7 pathway activation in CCRF-CEM (**A**,**B**) and K-562 (**C**,**D**) cells. Caspases proteolytic activity was measured by fluorescence of 485 nm/538 nm after 24 h (**A**,**C**) and 48 h (**B**,**D**) incubation with toxin. Values are means ± SD (*n* = 4). *** *p* < 0.001.

We also performed an analysis that focused on an examination of melittin effect on activation of the Caspase-3/7 pathway in human leukaemia cell lines. During the caspase’s activity estimation, we observed that treatment of both cell lines resulted in both a dose-dependent and time-dependent increase in 530 nm fluorescence (Figure 6), which clearly indicated increased proteolytic activity of caspase 3/7 in these cells.

## 4. Discussion

Leukaemia involves a set of malignant conditions that disturb blood and blood forming tissues. In leukaemia, normal haematopoiesis is suppressed by the uncontrolled proliferation and accumulation of leukemic cells [38]. Leukaemia has been classified into four major categories based on the types of cells affected and by the developmental stage of the originating cells. This division includes: acute myeloid (AML) and acute lymphoblastic leukaemia (ALL) as well as chronic myeloid (CML), and chronic lymphoblastic leukaemia (CLL) [39]. A statistical cancer analysis performed by Seigel et al. demonstrated that leukaemia represents around 3.5% of all the cancer cases estimated in the United States in 2019 [40]. The main clinical treatment for leukaemia is chemotherapy with substances that induce cellular apoptosis. The development of therapies aimed at leukaemia has progressed substantially in recent years but the disease still remains one of the most challenging and problematic type of cancers to treat successfully.

In this study, we performed analysis analyses on two different types of leukaemia cells: ALL and CML, which are one of the most serious and most common types of leukaemia.

ALL is the most common form of paediatric leukaemia and the second most common acute leukaemia in adults. This type of leukaemia is characterised by a malignant transformation of progenitor cells of lymphoid origin in blood forming tissues: bone marrow, blood and extramedullary sites. The estimated incidence of this type of leukaemia is about 1.6 per 100,000 population [41]. Treatment of adult ALL has been adjusted from paediatric ALL treatment protocols. Induction therapy aims to return haematopoiesis to normal function through complete remission, however, this is achieved only in up to 40% of adult patients [42].

CML is a clonal myeloproliferative disorder of hematopoietic stem cells (HSCs) that accounts for 15–20% of all cases of adult leukaemia. The molecular basis is a genetic translocation (9;22)(q34;q11) of the Abelson gene (ABL1) from chromosome 9q34 with the breakpoint cluster region (BCR) gene on chromosome 22q11.2. BCR–ABL1 transcripts have been found in over 90% of CML patients [43]. The BCR-ABL1 gene fusion encodes a constitutively expressed tyrosine kinase, which induces cell growth and replication via RAS, RAF, JUN kinase, MYC and STAT signalling pathways. The activation of a cytokine-independent cell cycle promotes tumorigenesis and abnormal signalling of apoptosis [44].

Cell viability and cell death are the two main parameters that are primarily used for a general assessment of anti-leukemic activity of tested chemical compounds, and these may provide new potential therapeutics [45]. Cell viability is highly dependent on the various processes of regulated cell death which can be inducted by numerous extracellular and intracellular signals [46]. In the presented study, we demonstrated the high antileukemic potential of melittin against two different types of leukaemia cell lines, CCRF-CEM and K-562, which have been confirmed by using two different cytotoxicity assays with trypan blue dye and in MTT test (Figure 2).

As an amphipathic helical peptide, melittin is able to incorporate itself into the phospholipid bilayers, resulting in the disruption of the cell membrane leading to intensive cytotoxicity of all cells treated by this toxin. This action was confirmed on the red blood cells, where melittin caused haemolysis [47]. For this reason, we decided to perform an analysis of the physiological cells which occur in blood flow and can be a healthy control to verify the cytotoxic effect. For this study, we used PMBCs obtained from healthy donors, which are very often used and suggested as a healthy control in leukaemia studies [48,49]. Our result demonstrates that the beneficial effect of melittin, which can act as a potential anti-leukemic agent, does not induce any negative changes (in cell viability and morphology) in PBMCs (Figure 3). This observation is in line with a study performed by Tu et al. [30] where melittin was able to induce apoptosis in human melanoma A2058 cell line but did not have any influence on normal skin fibroblast Detroit 551 cell line. This suggests that unphysiological cell lines are more sensitive to melittin action.

The anti-leukemic effects of melittin can follow different paths such as membrane disruption, energy metabolism modulation, inhibition of cell proliferation and cell death (apoptosis and necrosis) inducing [50]. One of the first studies demonstrating melittin’s cytotoxic action on tumour cells was performed in 1985, Hait et al. [15]. It demonstrated that melittin is able to inhibit calmodulin and thus competitively inhibits Ca^2+^ pump activity. This event results in an increase in Ca^2+^ concentration that is toxic to cells and eventually induces cell death. The study performed by Chu et al. [51] demonstrated that melittin induced an increase in the intracellular Ca^2+^ concentration through activation of L-type Ca^2+^ channels, while Ryu et al. [52] showed the effect of melittin on intracellular Ca^2+^ mobilization and exocytosis via different independent signalling pathways. All these studies confirmed that melittin mediated the increase of Ca^2+^ concentration but the mechanism needs to be clearly confirmed in all cases.

Apoptosis takes place as a physiological state of the organism during development and ageing as well as a homeostatic mechanism to maintain cell populations in different tissues. This process is also initiated during activation of defence mechanisms such as immune responses or when cells are damaged by different factors. During the apoptotic process, there is no inflammatory reaction. This is related to three main characteristics of apoptosis: Apoptotic cells do not release their cellular ingredients into the extracellular matrix; the cell fragments are rapidly phagocytosed by neighbouring cells and, the engulfing cells do not produce anti-inflammatory cytokines [53]. For these reasons, the benefit for the organism is to direct non-physiological cells to apoptosis and the new potential anti-leukaemia agents must induce this process. For this reason, we decided to determine the potential mechanism related to apoptosis of leukaemia cell death induced by melittin, which we observed in trypan blue dye and in MTT tests (Figure 2).

A characteristic event of the apoptotic process is the expression of phosphatidylserine (PS) surface biomarkers, on the cellular membrane lipid bilayer, which leads to the recognition of apoptotic cells for phagocytosis [54]. In our study we evaluated the presence of PS on melittin treated cells using a kit with recombinant phosphatidylserine-binding protein, Annexin V, which is able to strongly and specifically interact with phosphatidylserine residues [55,56]. Our flow cytometry analysis clearly demonstrated the presence of PS on cells treated by melittin, which confirms the induction of apoptosis by this compound (Figure 5).

The mechanisms of apoptotic cell death are extremely complex and sophisticated, involving an energy-dependent cascade of various molecular events. The apoptosis can be triggered through two main pathways, namely the extrinsic/receptor and intrinsic/mitochondrial pathways. However, the majority of chemotherapeutic agents are responsible for activation of the intrinsic pathways. This pathway of apoptosis uses the mitochondria and mitochondrial proteins to mitochondrial outer membrane permeabilization (MOMP). Intermembrane proteins like cytochrome c, second mitochondria-derived activator of caspase (SMAC) and Omi are released following MOMP. Upon the release of cytochrome c, the apoptosome is formed [57]. The mitochondrial membrane potential (ΔΨm) generated by proton pumps is a major component in the process of energy storage during the process of oxidative phosphorylation. The decrease of ΔΨm is very often an early event in the apoptotic process related to the mitochondrial pathway. The loss of ΔΨm may not be an early requirement for apoptosis but may be a consequence of the apoptotic-signalling pathway that depended on the mitochondrial pathway [58]. In our study, we demonstrated a dose dependent decrease of ΔΨm in both cellular models treated by melittin, which gave us confirmation of the apoptosis process and suggested a potential pathway related to the intrinsic mechanism that depended on mitochondria (Figure 4).

The major elements of the molecular mechanism of apoptosis are enzymes called caspases. These protease enzymes cleave proteins at positions containing aspartic acid residues. The specificity of different caspases is dependent on recognition of neighbouring amino acids. Furthermore, activation of caspases appears to lead to an irreversible induction of programmed cell death. Caspases have been divided according to their physiological roles. Into initiators (caspase-2,-8,-9,-10), effectors or executioners (caspase-3,-6,-7), as well as inflammatory caspases (caspase-1,-4,-5) [53]. To confirm our theory of the mitochondria-dependent mechanism of apoptosis in leukaemia cells based on flow cytometry PS estimation and ΔΨm measurements, we decided that the last phase of our large-scale study about the antileukemic potential of melittin should be an evaluation of the ability of melittin to activate caspases 3 and 7. The results obtained have clearly confirmed apoptosis induction in both cell lines (Figure 6).

Our results confirm the study of Lee et al. [59] that melittin is involved in the mitochondria-and caspase-dependent apoptotic pathway. They showed that melittin disrupts ΔΨm and induces the Ca^2+^ release from the endoplasmic reticulum and its remarkable accumulation in mitochondria.

Of all the concentrations that were demonstrated and that proved to be effective during the course of our study, measurements above 1 µM correspond to another study conducted by Park et al. [20] which found that melittin at a concentration of 1.05 µM inhibits human renal carcinoma invasion. In other studies, melittin at a concentration of around 0.9 µM induced apoptosis in prostatic carcinoma cells [21]. Melittin at a concentration of 0.7 µM induced apoptosis and thereby inhibited ovarian cancer cell growth [22]. Finally, Jeong et al. [27] also demonstrated that melittin at a concentration of 0.7 µM significantly inhibited cell invasion and migration in breast cancer cells.

## 5. Conclusions

We have demonstrated for the first time that melittin is able to induce the apoptosis process via the intrinsic/mitochondrial pathway in different models of leukaemia. This is a very important step for research as a new potential anti-leukaemia as well as anticancer therapy. Further analysis on a molecular level has been planned for our next research project, which will focus also on melittin.

## Figures and Tables

**Table 1:** Examples of various biological effects of melittin.

The Biological Effect of Melittin	Reference
Inhibits clonogenicity and growth in leukemic cells of humans and mice	[15]
Activates of secretory phospholipase A2	[17]
Demonstrates antibacterial activity against strain of *Staphylococcus aureus* (strain 80) resistant to penicillin	[18]
Inhibits invasion and migration of non-small cell lung cancer cells induced by the epidermal growth factor	[19]
Inhibits human renal carcinoma invasion	[20]
Inhibits growth of human prostate cancer cells	[21]
Induces apoptotic cell death in ovarian cancer cells	[22]
Inhibits proliferation and induction of apoptosis in malignant human glioma cells.	[23]
Inhibits proliferation and induction of apoptosis in human hepatocellular carcinoma cells	[24,25,26]
Inhibits breast cancer cell invasion and migration	[27]
Inhibits human cervical cancer progression and angiogenesis.	[28]
Inhibits proliferation of the osteosarcoma cells	[29]
Induces apoptosis in human melanoma cells	[30]
Exerts tachycardic effects by activating COX pathway	[31]
Increasing blood pressure and reversing hypotension in haemorrhagic shock	[32]

## Supplementary Materials

The following are available online at https://www.mdpi.com/2218-273X/10/2/247/s1, Figure S1: Gating strategies during flow cytometric analysis. Gates established on unlabelled cells.
