# Supplementary material for: Melittin—A Natural Peptide from Bee Venom Which Induces Apoptosis in Human Leukaemia Cells
Source: Biomolecules. 2020 Feb 6;10(2):247. doi: 10.3390/biom10020247 (PMC7072249; doi:10.3390/biom10020247)
Supplement: Supplementary file 1 [file biomolecules-10-00247-s001.pdf]

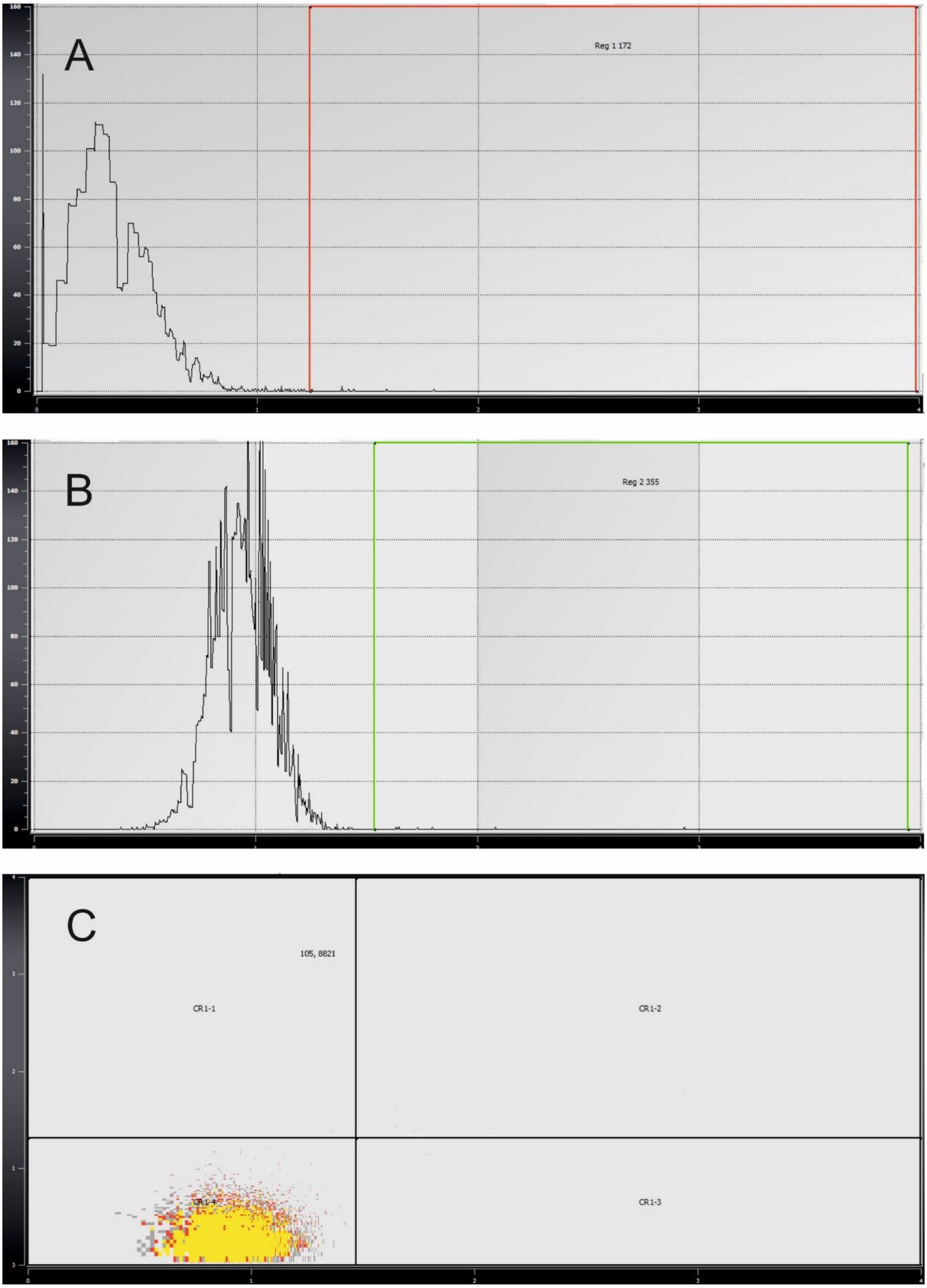

Figure S1. Gating strategies during flow cytometric analysis. Gates established on unlabelled cells. The figure is presenting selected analysis for the control sample. A – histogram for PI analysis; B – histogram for FITC; C – dot plot where x is FITC channel and y is the channel for PI. Pictures were generated from the flow cytometer PARTEC CUBE 6 (Görlitz, Germany) CyFlow software version 1.5.1.2.
